# Classification of Patients for Whom Benefit of Long-term Opioid Therapy No Longer Outweighs Harm: Protocol for a Delphi Study

**DOI:** 10.2196/33310

**Published:** 2022-03-04

**Authors:** Raymond Van Cleve, Sara Edmond, Jennifer Snow, Anne C Black, Jamie L Pomeranz, William Becker

**Affiliations:** 1 Center for Innovation to Implementation Menlo Park, CA United States; 2 Stanford University Palo Alto, CA United States; 3 Department of Psychiatry Yale School of Medicine New Haven, CT United States; 4 Pain Research, Informatics, Multimorbidities, and Education Center VA Connecticut Healthcare System New Haven, CT United States; 5 Department of Internal Medicine Yale School of Medicine New Haven, CT United States; 6 Department of Occupational Therapy University of Florida Gainsville, FL United States

**Keywords:** modified Delphi technique, long-term opioid treatment, chronic pain, opioid therapy, opioids, pain management, Delphi study

## Abstract

**Background:**

Patients with chronic pain prescribed long-term opioid therapy may come to a point where the benefits of the therapy are outweighed by the risks and tapering is indicated. At the 2019 Veterans Health Administration State of the Art Conference, there was an acknowledgment of a lack of clinical guidance with regard to treating this subset of patients. Some of the participants believed clinicians and patients would both benefit from a new diagnostic entity describing this situation.

**Objective:**

The aim of this study was to determine if a new diagnostic entity was needed and what the criteria of the diagnostic entity would be. Given the ability of the Delphi method to synthesize input from a broad range of experts, we felt this technique was the most appropriate for this study.

**Methods:**

We designed a modified Delphi technique involving 3 rounds. The first round is a series of open-ended questions asking about the necessity of this diagnostic entity, how this condition is different from opioid use disorder, and what its possible diagnostic criteria would be. After synthesizing the responses collected, a second round will be conducted to ask participants to rate the different responses offered by their peers. These ratings will be collected and analyzed, and will generate a preliminary definition for this clinical phenomena. In the third round, we will circulate this definition with the aim of achieving consensus.

**Results:**

The modified Delphi study was initiated in July of 2020 and analysis is currently underway.

**Conclusions:**

This protocol has been approved by the Internal Review Board at the Connecticut Veterans Affairs and the study is in process. This protocol may assist other researchers conducting similar studies.

**International Registered Report Identifier (IRRID):**

DERR1-10.2196/33310

## Introduction

### Background

Although the number of opioid prescriptions has decreased since 2012 [1], long-term opioid therapy (LTOT) remains a common treatment for chronic pain [2], with the duration of therapy potentially lasting for years [3,4]. Patients prescribed LTOT for pain are at risk for adverse outcomes, including worsening pain and function and developing opioid use disorder (OUD) [5]. A challenge in clinical, research, and policy spheres is determining whether and how to apply the Diagnostic and Statistical Manual of Mental Disorders, Fifth Edition (DSM-5)’s OUD criteria to patients receiving LTOT for whom benefit is no longer outweighing harm and tapering is thus indicated per consensus guidelines [6-8]. The DSM-5 criteria for OUD are designed to identify a condition in which patients have compulsive use of opioids leading to adverse consequences and do not necessarily have comorbid chronic pain [9].

Thus, some experts have argued that a new diagnostic entity specifically intended for patients on LTOT for whom harm is outweighing benefit would help advance research and clinical care. These experts believe concurrent opioid dependence and chronic pain are more than the sum of their parts, and a new diagnostic entity could address this complexity [10], something clinicians and researchers have struggled with in using the OUD diagnosis on its own. During the 2019 Veterans Health Administration (VHA) Health Services Research and Development (HSR&D) State of the Art (SOTA) Conference, consensus emerged on the need for a Delphi study to understand if a new diagnostic entity is needed and, if so, to develop consensus on its criteria and characteristics [11]. The modified Delphi method is designed to gain consensus on a discrete topic that does not yet have a clear definition [12] and has been used in previous studies concerning OUD. Given the problem articulated during the SOTA Conference, this method lends itself well to investigating a problem of this complexity [13,14]. The aim of this paper is to describe the protocol for a Delphi study to explore the need for and criteria of a new diagnostic entity characterizing the clinical scenario of benefit no longer outweighing harm of LTOT for chronic pain.

### Study Objectives

The objectives of this Delphi study are to (1) explore perspectives on the merits of creating a new diagnostic entity, separate from but not replacing OUD, that better characterizes the scenario of benefits no longer outweighing harm of LTOT for chronic pain and (2) develop consensus on its definition and diagnostic criteria. We will present questions in an open-ended format to avoid bias and allow the responses of the participants to guide the analysis.

## Methods

### Overview

The Delphi method is well suited for surveying expert panels to gain consensus on a clinical problem that is not well defined and determine the defining elements of that problem. Consistent with other related studies, we will conduct the Delphi study remotely to promote independent contribution and avoid “bandwagon” or “halo” effects, and generate as many ideas as possible from each individual contributor [15]. The halo effect refers to an instance where a positive response given by a member of a panel influences subsequent responses [16] and the bandwagon effect is when one theme identified by a member of a panel alters subsequent responses [17]. Given the structure of the Delphi method, we were not concerned about these biases occurring.

**Figure 1:**
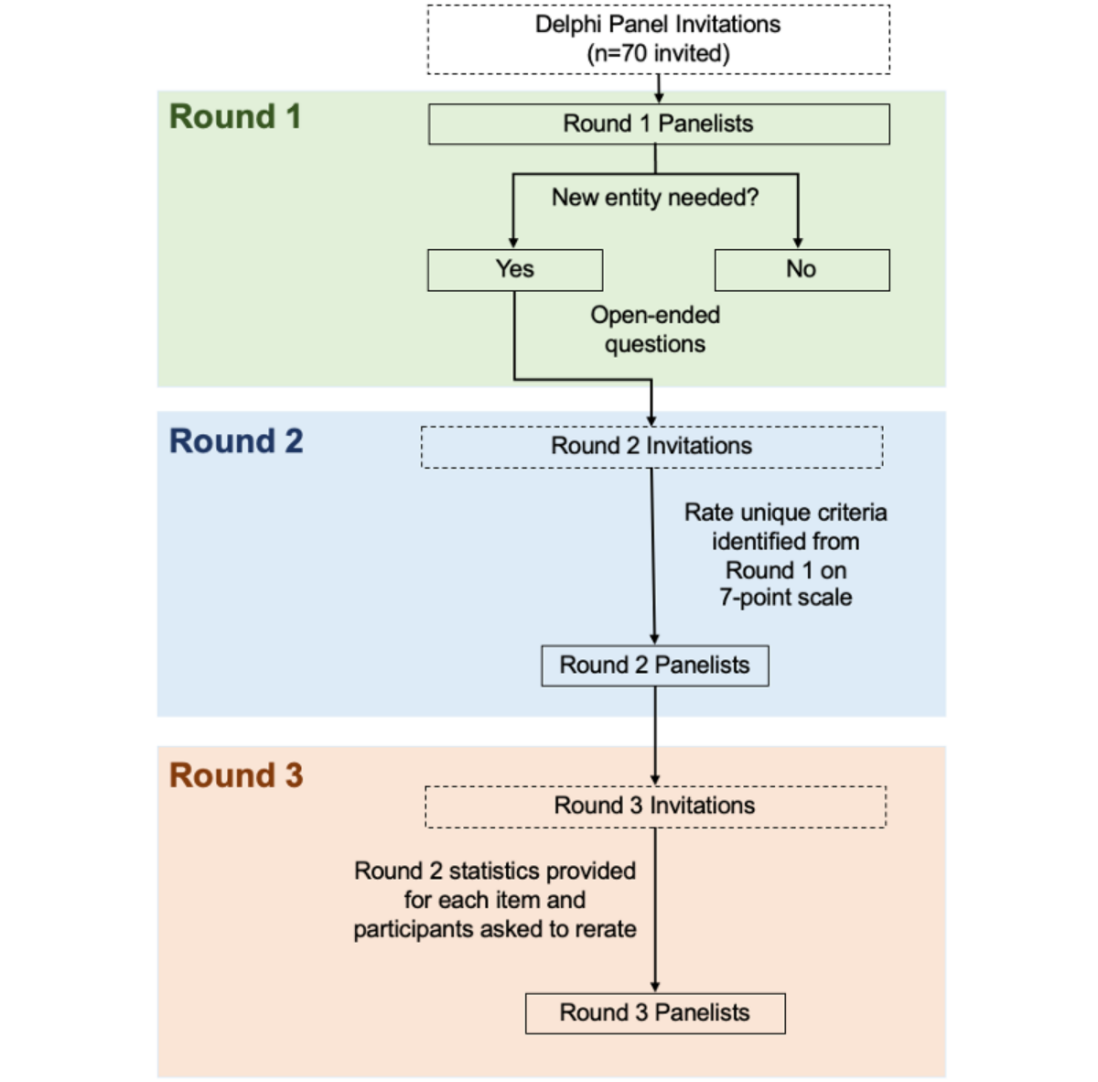
CONSORT flow diagram for a 3-round Delphi study.

The Delphi study flow is shown in Figure 1. The first round of this Delphi process will have a screening question, described below, and will then elicit responses to open-ended questions on the potential new diagnostic entity. Once these responses are collected, they will be analyzed using rapid qualitative matrix and content analysis [18-20]. Results from the qualitative analysis will summarize expert-proposed diagnostic criteria that will be evaluated using numeric rating scales in round 2. Round 2 group statistics summarizing ratings of each proposed criterion (ie, means, standard deviation, the medians, and interquartile ranges) will be presented to the expert panel in round 3, when experts will be asked to review their previous ratings relative to the measures of central tendency, and to re-evaluate each item. Results from the third round will be summarized in accordance with the consensus criteria originally established prior to the initiation of the Delphi study.

### Selection of Delphi Participants and Inclusion Criteria

As above, VHA HSR&D convened a 2-day SOTA Conference on effective pain management and opioid safety in September 2019. The purpose of the SOTA Conference was to convene a multidisciplinary group of experts to help HSR&D develop specific research priorities related to increasing access to medications for OUD, managing LTOT for pain including tapering, and treating co-occurring pain and substance use disorders [21]. To ensure as robust a process as possible, HSR&D invited a diverse array of subject matter experts from within and outside the VHA with expertise in pain, OUD, opioid safety, and clinical research representing the following disciplines: general internal medicine, psychology, addiction medicine, addiction psychiatry, nursing, pharmacy, pain medicine, neurology, clinical epidemiology, health services research, and health policy. We will recruit participants for the Delphi study from the list of invitees to the SOTA Conference (N=70). We explicitly chose to recruit invitees rather than participants in the SOTA Conference as we did not want to exclude the perspectives of experts who arbitrarily were unavailable to attend the SOTA Conference.

We set a date for the completion of each round of the Delphi study and will send out regular reminders to participants who have not completed that round to try to minimize attrition. We do not anticipate significant attrition given that many of the participants are already collaborating on similar projects and have voiced an interest in participating in this type of study. However, if we observe significant attrition (eg, >20%), we will consider alternative data collection methods such as offering to conduct data collection via phone.

### Delphi Process

#### Overview

All 3 rounds of the survey will be administered by Qualtrics XM (Qualtrics International Inc), which has been licensed to our research team through Yale University. We will send individual emails to participants explaining the Delphi process and why they were selected to participate. In the email, we will acknowledge the discussion at the 2019 VHA HSR&D SOTA Conference that prompted this study. The survey will contain a link to the DSM-5 criteria for OUD for participant reference. We will allow approximately 1 month to complete each round, with some reminder emails if necessary.

#### Round 1

##### Aim

The aims of round 1 are as follows: (1) to understand the various expert perspectives on the merits of creating a separate diagnostic entity and (2) to gather information about the defining characteristics of the clinical phenomenon requiring operationalization, as well as information about how the phenomenon relates to OUD via several open-ended questions. We designed a survey that will take approximately 25-30 minutes to complete. Questions such as “Please describe a person who would be diagnosed with Condition X” are meant to prime participants to envision how these patients might present in a clinical setting. We also wanted to encourage participants to consider the bounds of this condition, defining both what it is and what it is not.

##### Data Collection

The round 1 survey can be found in Textbox 1. To meet aim 1, we will use the question, “Do you think a new diagnostic entity is needed for patients who have been taking opioids and for whom the potential harms of the therapy outweigh the benefits of the therapy?” as a screening question; only participants who answer “yes” to this question will be invited to complete the rest of the survey. As shown in Textbox 1, the remainder of round 1 items are a mix of yes/no and free-text questions.

**Questions included in round 1 of this Delphi study.**

Note: Questions 2-9 were only asked of those who said “yes” to question 1.

1. Do you think a new diagnostic entity is needed for patients on long-term opioid therapy for pain that is related to but distinct from the Diagnostic and Statistical Manual of Mental Disorders, Fifth Edition’s opioid use disorder definition? (yes/no)
  If yes, please explain why. If no, please explain why not.
2. Please describe a person who would be diagnosed with Condition X (How did he or she present? What were they prescribed? How did the treatment course go? What behaviors manifest themselves over time? How is this person different from a person with opioid use disorder)?
3. How would you differentiate Condition X from opioid use disorder?
4. Please complete the sentence “Condition X is defined as ____”
5. Please list the diagnostic criteria for Condition X:
6. Should Condition X have different gradations (eg, mild, medium, severe)? (yes/no)
  If yes, what gradations would you recommend? How would you distinguish between the different gradations?
  If no, why not?
7. Is Condition X related to opioid use disorder? (yes/no)
  If yes, how so?
8. What are the differences in how Condition X should be treated compared to opioid use disorder?
9. We have been using the term Condition X as a placeholder for ease of discussion. What do you think this condition should be called?

##### Data Analysis

We will use rapid qualitative analysis [20] to summarize free-text, open-ended answers. Summaries of individual responses will be inputted into a data matrix that will be used to identify distinct concepts within each section of the survey. We will then perform a content analysis to assess the concentration of each concept and generate a list of potential diagnostic criteria. The list of potential diagnostic criteria will be the basis for the second round. We will only include criteria mentioned by at least two Delphi participants in the round 2 survey.

#### Round 2

##### Aim

The objective of the second round will be to begin building consensus on the potential criteria defining the proposed new diagnostic entity. We will ask the participants to evaluate and rate the relevance of each criterion to the diagnostic entity. The experts will have the opportunity to critique the initial responses (that have been analyzed and reformatted) and suggest the criteria that should be retained.

##### Data Collection

The round 2 survey will list each potential criterion and ask participants to answer, “to what extent do you agree that each of the following features/criteria should be included as a feature/criterion of Condition X?” Participants will answer on a 7-point Likert scale from “strongly disagree” to “strongly agree.” We will also ask participants to comment on their preferred wording among alternatives, and provide suggestions for alternative wording for diagnostic criteria. Finally, we will ask participants to indicate their favored proposed names for the new diagnostic entity.

##### Data Analysis

For each item, we will calculate the mean, standard deviation, median, and interquartile range of ratings. We agreed a priori that diagnostic criteria with a median of 5 or greater will indicate consensus for inclusion, median scores between 3 and 5 will indicate the need for further exploration, and a median score below 3 will indicate that an item should not be included. We will use rapid qualitative analysis to explore the qualitative feedback. Qualitative feedback will be used to identify any additional potential criteria for inclusion in round 3.

#### Round 3

##### Aim

This final round will ask panelists to rerate potential criteria in the context of central tendency statistics in an effort to generate further consensus.

##### Data Collection

We will present to the group the mean, standard deviation, median, and interquartile range of ratings for each proposed criterion from round 2, along with that panelist’s initial response on the 7-point Likert scale. Participants will be asked to rerate each item from round 2 and provide initial ratings for any additional items identified based on round 2 qualitative feedback.

##### Data Analysis

Consistent with prior Delphi studies, items with a median of 5 or greater after round 3 will be recommended for inclusion in the criteria for the new diagnostic entity.

### Ethics Approval

This protocol was approved by the Institutional Review Board of VA Connecticut Healthcare System (approval number: 1624733-3).

## Results

Data collection for round 1 began in July 2020; we have completed the first two rounds and are currently collecting round 3 data. We plan to publish qualitative findings from round 1 and overall findings from the entire study.

## Discussion

Herein, we describe a protocol for a Delphi study designed to develop criteria to characterize the phenomenon experienced by patients with chronic pain prescribed LTOT when benefits of the therapy no longer outweigh harms. The protocol uses rigorous methods to iteratively generate consensus among a diverse group of subject matter experts; as such, it holds promise in providing momentum forward on a topic that has become increasingly relevant during the United States’ efforts to improve the safety of opioid prescribing, including tapering when harm outweighs benefit.

Criteria for the novel diagnostic entity may be used to standardize the definition of the new entity, promote targeted research, inform clinical practice guidelines for its treatment, and ultimately improve quality of care. The Delphi method provides the process to accomplish this goal by iteratively developing expert consensus regarding the definition of the entity and a preliminary list of its diagnostic criteria.

If consensus is achieved around a definition and list of criteria for a new diagnostic entity, the next steps may include convening a work group to refine the criteria and evaluating the criteria in practice with regard to sensitivity and specificity. Identification of an entity distinct from OUD could inform practice specific to that entity. Future work will include exploring best pathways for disseminating our findings to frontline clinicians who work with patients prescribed LTOT, and determining how to use our findings to inform future research, policy work, and clinical care, such as developing better treatments for patients with chronic pain prescribed LTOT and helping frontline clinicians assess and provide tailored treatment options.

## Abbreviations

DSM-5: Diagnostic and Statistical Manual of Mental Disorders, Fifth Edition
HSR&D: Health Services Research and Development
LTOT: long-term opioid therapy
OUD: opioid use disorder
SOTA: State of the Art
VHA: Veterans Health Administration
